# Streptococcus mutans exacerbates gut microbiota dysbiosis in SHANK3^**-/-**^ autism model mice via the oral-gut axis

**DOI:** 10.1080/20002297.2026.2681259

**Published:** 2026-06-04

**Authors:** Wenyan Huang, Shaohong Wang, Yan Zhang, Min Gao, Ning Zhong, Changhua Hao, Lal Pathak Janak, Lijing Wang, Si Meng, Wanghong Zhao, Sujuan Zeng

**Affiliations:** a Department of Pediatric dentistry, School and Hospital of Stomatology, Guangdong Engineering Research Center of Oral Restoration and Reconstruction & Guangzhou Key Laboratory of Basic and Applied Research of Oral Regenerative Medicine, Guangzhou Medical University, Guangzhou, China; b Department of Stomatology, Shanghai Pudong Hospital, Fudan University Pudong Medical Center, Shanghai, China; c Beijing University of Chinese Medicine Shenzhen Hospital (Longgang), Shenzhen, China; d Department of Stomatology, Nanfang Hospital, Southern Medical University, Guangzhou, Guangdong, China

**Keywords:** *Streptococcus mutans*, autism spectrum disorder, microbiota-gut-brain axis, oral-gut axis, metagenomics

## Abstract

**Background and objective:**

Autism spectrum disorder (ASD) is associated with gut microbiota dysbiosis, yet the impact of oral pathobiont translocation via the oral–gut axis remains unclear. This study investigated how *Streptococcus mutans* (*S. mutans*), a primary cariogenic pathogen, influences gut microbial structure and function in an ASD mouse model.

**Methods:**

SHANK3 knockout (SHANK3^-/-^) and wild-type (WT) mice were divided into four groups: WT control, WT *S. mutans*-gavaged (WT-S.m), SHANK3^-/-^ control, and SHANK3^-/-^ *S. mutans*-gavaged (SHANK3-S.m). Mice were gavaged with *S. mutans* UA159 twice weekly for five weeks, followed by fecal metagenomic sequencing (*n* = 6 per group).

**Results:**

*S. mutans* translocated to the gut in both gavaged groups but did not achieve enhanced colonization in SHANK3^-/-^ mice. *S. mutans* gavage significantly altered the gut microbiota structure in both WT and SHANK3^-/-^ mice. In the ASD model, *S. mutans* gavage led to a significant enrichment of potential pathobionts (e.g. *Duncaniella dubosii*, *Muribaculum gordoncarteri*) and a decrease in beneficial bacteria (e.g. *Bacteroides caecimuris, Bacteroides faecium*). LEfSe analysis identified *Parascardovia denticolens* and *Bacteroides heparinolyticus* as specific biomarkers for the SHANK3-S.m group. Microbial networks showed reduced stability in SHANK3-S.m mice, with *Enterocloster bolteae* as a key node. Functional analysis revealed suppressed butanoate metabolism and enhanced neuroinflammation-related pathways.

**Conclusion:**

Although *S. mutans* colonized only transiently, it provoked exacerbated ecological instability and pro-inflammatory metabolic alterations in ASD model mice, underscoring the role of the oral-gut-brain axis in ASD.

## Introduction

Autism spectrum disorder (ASD) is a complex neurodevelopmental condition characterised by core deficits in social communication, repetitive and stereotyped behaviours, and sensory abnormalities, which collectively lead to severe and long-term impairment of social functioning [1,2]. The global prevalence of ASD has been rising steadily. Recent 2022 data from the United States indicates a prevalence of 32.2 per 1,000 (approximately 1 in 31) among 8-year-old children [3]. Research in China corroborates this significant disease burden, showing a prevalence of 0.70% among children aged 6 to 12 years, with a significantly higher incidence in males than females (0.95% vs. 0.30%) [4]. The total number of individuals with ASD in China is estimated to exceed 10 million, with approximately 200,000 new cases reported annually [5]. Current clinical management of ASD primarily relies on behavioural therapy and pharmacological treatment of co-occurring symptoms; effective interventions capable of ameliorating the core symptoms remain elusive [6].

The aetiology of ASD involves a complex interplay between genetic predisposition and environmental factors. Dysfunction of the microbiota-gut-brain axis (MGBA) is strongly implicated in its pathophysiology [7–9]. Substantial evidence confirms significant differences in gut microbiota composition between individuals with ASD and those with typical development (TD), manifesting as overall dysbiosis and a reduction in beneficial bacteria [10–13]. Gut microbes can influence central nervous system function by modulating the MGBA through metabolic, immune, and neural pathways [8]. Clinical studies further indicate that ASD-associated gut microbial disturbances emerge early in childhood and coincide with the onset of behavioural symptoms [14]. Both animal and clinical trials have demonstrated that faecal microbiota transplantation from TD donors can alleviate gastrointestinal and behavioural symptoms in ASD models and human subjects, highlighting the therapeutic potential of microbiota-targeted interventions [15–17].

Gastrointestinal (GI) comorbidities are common in individuals with ASD, and the severity of GI symptoms is closely linked to the core behavioural abnormalities of ASD [18–21]. This association may be related to impaired intestinal barrier function. For instance, serum levels of zonulin, a marker of intestinal permeability, are significantly elevated in children with ASD and positively correlate with disease severity [22,23]. Studies have confirmed that restoring the gut barrier with probiotics, such as *Clostridium butyricum*, can improve behavioural deficits in ASD mouse models [24]. These findings highlight the crucial role of the gut microbiome in the pathogenesis of ASD. However, existing research has predominantly focused on indigenous gut microbiota, largely overlooking the potential pathological impact of oral microbes migrating to the gut via swallowing.

Dental caries, a classic example of oral microbial dysbiosis, is increasingly recognised for its association with systemic health. The primary cariogenic pathogen, *Streptococcus mutans* (*S. mutans*), is not only responsible for local cariogenesis but can also translocate to distant organs via hematogenous spread or daily swallowing, associating with systemic conditions like endocarditis and stroke [25–27]. Routine oral activities or dental procedures can facilitate bacterial entry into the bloodstream [28,29]. Furthermore, the daily swallowing of approximately 1.5 liters of saliva, containing about 1.5 × 10^12^ oral bacteria, provides a continuous route for oral microbes to reach the gastrointestinal tract [30]. Studies have confirmed that oral administration of streptococci can induce gut microbiota dysbiosis and intestinal inflammation in mice [31]. In patients with inflammatory bowel disease (IBD), oral-originating serotype k *S. mutans* has been shown to exacerbate colitis, even at low bacteremia levels (1 × 10^4^ CFU) [32]. Notably, individuals with ASD are at a significantly higher risk of dental caries compared to the TD population, due to behavioural challenges and medication side effects [33,34]. Emerging evidence further suggests that oral-gut microbial crosstalk may be altered in ASD. A study by Kong et al. revealed disrupted co-occurrence patterns between oral and gut microbial communities in individuals with ASD, indicating that oral microbes may influence gut ecology through swallowing or immune-mediated pathways [35]. This evidence suggests that *S. mutans* translocation via the oral-gut axis may play a pivotal role in ASD-related gut dysbiosis and behavioural abnormalities, although the precise mechanisms remain unclear.

This study employed SHANK3 (SH3 and multiple ankyrin repeat domains 3) knockout (SHANK3^-/-^) ASD model mice to simulate the in vivo migration of *S. mutans* via the oral-gut route using oral gavage. Integrated with metagenomic sequencing, we systematically analysed the impact of *S. mutans* colonisation on the structure and function of the gut microbial community. Our aim is to elucidate the role of *S. mutans* in ASD-associated gut dysbiosis, thereby providing a new perspective on the oral-gut axis mechanisms in ASD.

## Methods

### Experimental animals and ethics

All mice were housed in a specific pathogen-free (SPF) facility at Guangdong Huawai Testing Co., Ltd. [Facility Use License No.: SYXK (Yue) 2023-0249]. The housing environment was maintained under controlled conditions: a 12-hour light/dark cycle, temperature of 18–23°C, relative humidity of 40–60%, and an air exchange rate of 10–20 times per hour. Mice were housed with a maximum of five per cage and had ad libitum access to food and water. All experimental procedures were conducted in accordance with the guidelines for the ethical use of laboratory animals issued by the Ministry of Health of the People's Republic of China, aiming to minimise the number of animals used and any potential suffering. The study protocol was approved by the Animal Ethics Committee of Guangdong Huawai Testing Co., Ltd. (Ethical Approval No.: 202203008).

### *Construction and genotyping of SHANK3* ^*-/-*^ *mice*

The SHANK3^-/-^ mice were generated on a C57BL/6J background by targeting exons 4–9 via CRISPR/Cas9-mediated genome editing. Specifically, sgRNA designed against the SHANK3 locus was co-injected with Cas9 protein into fertilised eggs to produce F0 chimeric mice. These founder mice were then crossed with wild-type C57BL/6J mice to establish a stable SHANK3^+/-^ heterozygous line. SHANK3^-/-^ homozygous mice used in this study were obtained by intercrossing SHANK3^+/-^ heterozygotes. Genomic DNA was extracted from mouse tail tissue using an alkaline lysis method. Genotype identification was performed via polymerase chain reaction (PCR). The PCR reaction mixture (25 μL total volume) consisted of 12.5 μL of 2 × Taq Master Mix, 1 μL each of forward and reverse primers (10 μmol/L), approximately 100 ng of template DNA, and nuclease-free ddH₂O to bring the volume to 25 μL. The primer sequences used were: for m-SHANK3^-/-^, forward 5′-TAGGATGGACCTGGACTTCTGACTG-3′ and reverse 5′-TAGCCCAGACTAAACGACAGCAG-3′; and for m-SHANK3-WT, forward 5′-ATAGGTGAGAGTTATGGCTGTCCGTC-3′ and reverse 5′-TATCAGAGCTGGCCGACACTGT-3′. The PCR amplification protocol was: 95 °C for 5 min; followed by 20 cycles of 98 °C for 30 s, 65 °C for 30 s (decreasing by 0.5 °C per cycle), and 72 °C for 45 s; then 20 cycles of 98 °C for 30 s, 55 °C for 30 s, and 72 °C for 45 s; with a final extension at 72 °C for 5 min. PCR products were separated by 2% agarose gel electrophoresis (140 V, 25 min) and visualised under UV light for genotype determination.

### Behavioural tests

To validate the autism-like phenotypes in SHANK3^-/-^ mice, we sequentially conducted the open field test (OFT), elevated plus maze (EPM) test, and three-chamber social test. All behavioural tests were performed between 8:00 and 11:00 AM. Prior to testing, mice were acclimatised to the behavioural testing room for at least 48 hours. All apparatuses were thoroughly cleaned with 75% ethanol after each test session.

For OFT, each mouse was placed in the centre of a 40 cm × 40 cm open arena and allowed to explore freely for 10 minutes. The total distance travelled, movement velocity, number of entries into the centre zone, and time spent in the centre zone were recorded and analysed using EthoVision XT 14.0 software to assess locomotor activity and anxiety-like behaviour.

For EPM test, the maze consisted of two open arms (30 × 5 × 0.5 cm), two enclosed arms (30 × 5 × 15 cm), and a central platform (5 × 5 cm), elevated 50 cm above the floor. Mice were placed in the central area facing an open arm and allowed to explore for 5 minutes. The number of entries into and time spent in the open and closed arms were recorded. The percentage of open arm entries and percentage of time spent in the open arms were calculated to quantify anxiety-like behaviour.

For three-chamber social test, the apparatus was a transparent box divided into three interconnected chambers. The test began with a 10-minute habituation phase, where the test mouse could freely explore all three chambers from the centre. In Phase 1 (sociability test), an unfamiliar wild-type (WT) mouse (Stranger 1) was enclosed in a wire cup in one side chamber, while the opposite chamber contained an empty cup. The test mouse was allowed to explore for 10 minutes. In Phase 2 (social novelty preference test), a second unfamiliar WT mouse (Stranger 2) was placed in the previously empty cup. The test mouse again explored for 10 minutes. The time spent interacting with each cup (containing a mouse or empty) was recorded to assess sociability and preference for social novelty.

### S. mutans culture and gavage protocol

The standard strain *S. mutans* UA159 was purchased from Hongce Biotechnology Co., Ltd. (Hangzhou, China). The bacteria were revived and subcultured in Brain Heart Infusion (BHI) broth under anaerobic conditions (80% N₂, 10% CO₂, 10% H₂) at 37 °C. Purity was confirmed by Gram staining and 16S rRNA gene sequencing. Bacteria from the logarithmic growth phase were harvested, resuspended in sterile physiological saline, and adjusted to a concentration of 1 × 10⁹ CFU/mL.

SHANK3^-/-^ mice and their WT littermates, aged 6-7 weeks, were randomly assigned to four groups (*n* = 10 per group): WT control (WT-C), WT *S. mutans*-gavaged (WT-S.m), SHANK3^-/-^ control (SHANK3-C), and SHANK3^-/-^ *S. mutans*-gavaged (SHANK3-S.m). Mice in the gavage groups received 100 μL of bacterial suspension (containing 1 × 10⁸ CFU S. mutans) by oral gavage twice weekly for five weeks. Control groups received an equal volume of sterile saline via the same route. All mice had free access to diet throughout the intervention period. A schematic diagram of the experimental workflow is presented in Figure 2A. At the end of the intervention, faecal samples (*n* = 6 per group) were collected from all mice and stored at −80 °C for subsequent metagenomic sequencing analysis.

### Metagenomic sequencing and bioinformatic analysis

Genomic DNA was extracted from faecal samples using the CTAB method. DNA concentration, integrity, and purity were assessed using an Agilent 5400 system. Sequencing libraries were prepared using the NEBNext® Ultra™ DNA Library Prep Kit for Illumina and sequenced on an Illumina NovaSeq platform with a PE150 strategy.

Raw reads were subjected to quality control as follows: adaptor sequences and low-quality reads were removed using Kneaddata, and host-derived reads were filtered out by alignment to the mouse reference genome using Bowtie2, resulting in high-quality clean reads. Taxonomic profiling was performed using Kraken2, and relative species abundances were estimated with Bracken. Functional annotation was carried out using HUMAnN3 against the UniRef90 database, and pathway abundances, including KEGG pathways, were obtained.

### In vivo imaging of bacterial translocation

In a separate experiment using a dextran sulphate sodium salt (DSS)-induced colitis model in BALB/c mice, we tracked the translocation and persistence of a luciferase-tagged *S. mutans* strain (Luc-S.m) via *in vivo* imaging. The results of this experiment are not included in the main figures but are available upon request.

### Statistical analysis

Measurement data conforming to a normal distribution are presented as mean ± standard deviation, while non-normally distributed data are presented as median and interquartile range. Differences in species or functional pathway abundances between groups were analysed using the Kruskal-Wallis test. Linear discriminant analysis Effect Size (LEfSe) was employed to identify differentially abundant biomarkers between groups. All statistical analyses were performed using R software. A *P*-value of less than 0.05 was considered statistically significant.

## Results

### *SHANK3* ^*-/-*^ *model exhibited core autism-like behavioural phenotypes*

**Figure 1.**
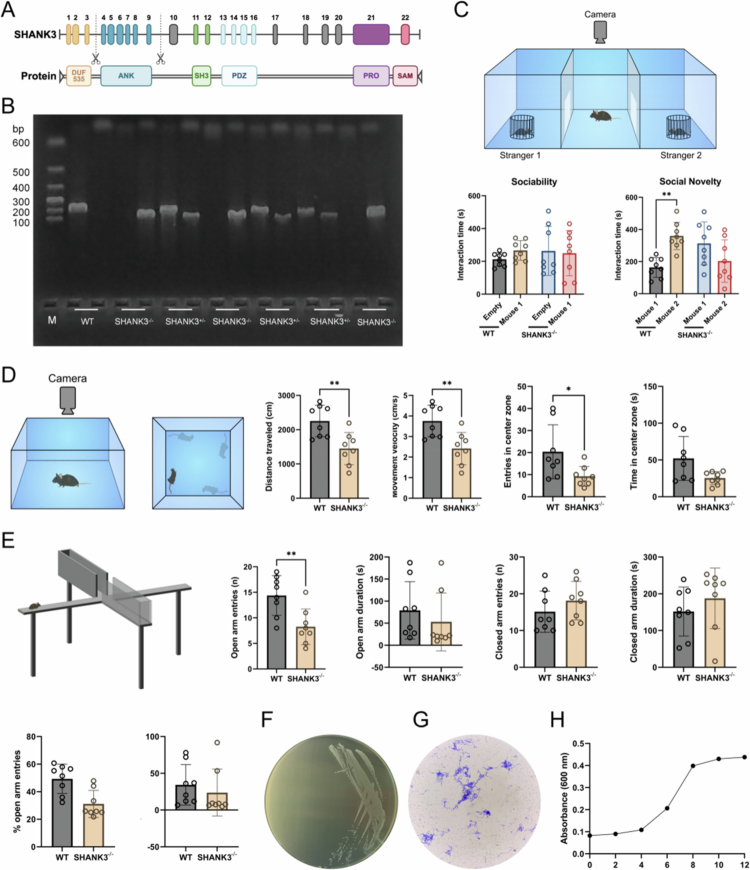
Construction and validation of the SHANK3^-/-^mouse model and characterisation of the *S. mutans* strain. (A) Schematic diagram of the SHANK3 gene targeting strategy, showing the deletion of exons 4–9. (B) Genotypic identification of SHANK3 knockout mice by PCR. Wild-type (WT), heterozygous (SHANK3^+/-^), and homozygous (SHANK3^-/-^) mice show specific bands at 260 bp, 260/175 bp, and 175 bp, respectively. *M* DNA marker. (C) Social novelty preference test in the three-chamber social paradigm. SHANK3^-/-^ mice spent significantly less time interacting with the novel stranger mouse (Stranger 2) compared to WT controls. Data are presented as mean ± SEM; ^**^ *P* < 0.01. (D) Open field test assessing anxiety-like behaviour. SHANK3^-/-^ mice exhibited reduced distance travelled and decreased activity in the centre zone. Data are presented as mean ± SEM; ^*^ *P* < 0.05, ^**^ *P* < 0.01. (E) Elevated plus maze test. SHANK3^-/-^ mice spent less time in the open arms, indicating increased anxiety-like behaviour. Data are presented as mean ± SEM; ^**^ *P* < 0.01. (F) Representative image of *S. mutans* UA159 colonies on a BHI agar plate after 10 hours of anaerobic culture. (G) Gram staining of *S. mutans* UA159, showing Gram-positive cocci in short chains (scale bar, 10 μm). (H) Growth curve of *S. mutans* UA159 in BHI broth under anaerobic conditions, showing the logarithmic (4–8 h) and stationary (8–12 h) phases.

An ASD mouse model was created through SHANK3 knockout (exons 4–9), with genotyping confirming the successful gene deletion (Figure 1A-B). In the three-chamber social test, during the social novelty phase, WT mice spent significantly more time interacting with the novel stranger mouse (Stranger 2) than with the familiar mouse (Stranger 1) (*P* < 0.05). In contrast, SHANK3^-/-^ mice spent more time with Stranger 1 than with Stranger 2, demonstrating a lack of social novelty preference (Figure 1C). SHANK3^-/-^ mice exhibited significantly increased anxiety-like behaviours, demonstrated by reduced time spent in and frequency of entries into the centre zone in the OFT, alongside increased entries into and time spent in the closed arms of the EPM (*P* < 0.05, Figure 1D-E).

### S. mutans gavage did not induce significant systemic toxicity in mice

Culture on BHI agar plates, Gram staining, and growth curve analysis confirmed that the standard strain *S. mutans* UA159 exhibited robust growth and typical morphological characteristics (Figure 1F-H). 16S rRNA gene sequencing analysis showed 99% similarity to the reference standard strain (Supplementary Table 1), confirming the accurate identity of the bacterial species.

**Figure 2.**
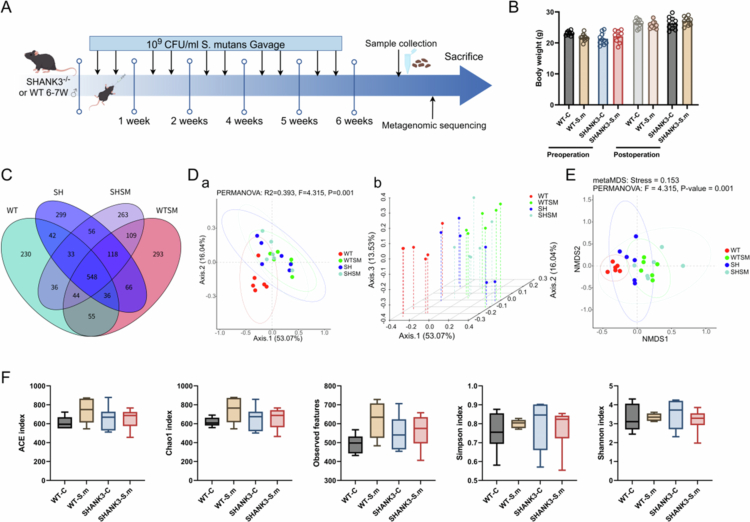
Effects of *S. mutans* gavage on body weight and gut microbiota structure in mice. (A) Schematic diagram of the experimental timeline for *S. mutans* administration and sample collection. (B) Body weight changes of mice in the four experimental groups after 5 weeks of intervention. Data are presented as mean ± SEM (*n* = 10 per group). (C) Venn diagram illustrating the number of unique and shared bacterial species at the species level among the four groups. (D) Principal coordinate analysis (PCoA) based on Bray-Curtis distance, showing the overall structural differences in gut microbiota. (a) 2D plot; (b) 3D plot. Percentages indicate the variance explained by each principal coordinate. (E) Non-metric multidimensional scaling (NMDS) plot based on Bray-Curtis distance (stress = 0.158). (F) Alpha diversity of gut microbiota across groups, as measured by the ACE, Chao1, Observed features, Simpson, and Shannon indices. Data are presented as mean ± SEM.

As shown in Figure 2B, body weight remained comparable across all groups during the intervention (*P* > 0.05), and all animals displayed normal clinical signs, including healthy fur and normal activity levels.

### S. mutans gavage alters gut microbiota structure in ASD model mice

Metagenomic sequencing of 24 mouse faecal samples yielded a total of 672,887,406 raw reads. After stringent quality control, 619,009,268 high-quality clean reads were obtained. The Q20 and Q30 scores for all samples exceeded 99% and 96%, respectively, indicating high sequencing data quality suitable for subsequent in-depth analysis (Supplementary Table 2).

Principal coordinate analysis (PCoA) based on Bray Curtis distance demonstrated that samples from the WT-C group clustered tightly, whereas those from SHANK3-C group and *S. mutans*-treated groups (WT-S.m, SHANK3-S.m) exhibited greater dispersion (Figure 2D). Notably, the WT-C group clustered separately from the other three groups, indicating that both SHANK3 knockout and *S. mutans* intervention significantly altered microbial community composition. PERMANOVA further confirmed significant intergroup differences (R² = 0.393, F = 4.319, *P* = 0.001). NMDS analysis (Figure 2E) additionally highlighted clear separations between WT-C and SHANK3-C, WT-C and WT-S.m, as well as SHANK3-C and SHANK3-S.m groups. Interestingly, *S. mutans* treatment led to convergent clustering of WT-S.m and SHANK3-S.m samples.

Alpha diversity indices (ACE, Chao1, Simpson, Shannon index, Observed features) showed no significant differences among the four groups (Figure 2F), suggesting that the interventions primarily affected microbiota composition rather than overall species richness and evenness.

### S. mutans gavage reshapes species composition in ASD model mice

Analysis of species composition, visualised by a Venn diagram (Figure 2C), revealed 548 species common to all four groups, while each group also possessed unique species (WT-C: 230; WT-S.m: 293; SHANK3-C: 299; SHANK3-S.m: 263).

**Figure 3.**
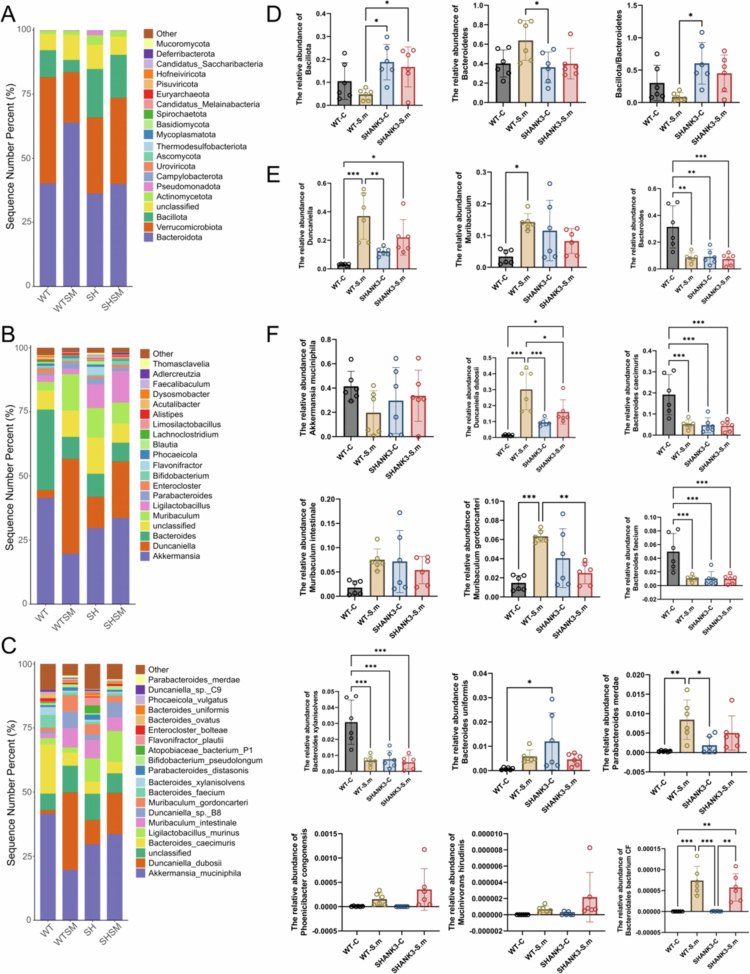
Gut microbiota composition across taxonomic levels following *S. mutans* intervention. Gut microbiota composition across taxonomic levels following *S. mutans* intervention. Bar plots display the mean relative abundance of dominant bacterial phyla (A), genera (B), and species (C) across the four experimental groups. The relative abundance and distribution of dominant bacterial phyla (D) and genera (E) across the four experimental groups are shown. (F) Statistical comparisons of key bacterial species among the four groups. ^*^ *P* < 0.05, ^**^ *P* < 0.01, ^***^ *P* < 0.001.

At the phylum level, Bacteroidota, Verrucomicrobiota and Bacillota were dominant in all groups. Compared to the WT-C and WT-S.m groups, the relative abundance of Bacillota was significantly increased in the SHANK3-C and SHANK3-S.m groups (*P* < 0.05, Figure 3A,D).

More pronounced changes were observed at the genus and species levels. Compared to the WT-C group, the abundances of *Duncaniella* and *Muribaculum* were significantly increased, while the abundance of *Bacteroides* was significantly decreased in the WT-S.m, SHANK3-C, and SHANK3-S.m groups (*P* < 0.05, Figure 3B,E). The abundance of *Duncaniella* was significantly elevated in the WT-S.m group relative to both the WT-C and SHANK3-C groups (*P* < 0.05), with a similar, though non-significant, trend observed in the SHANK3-S.m group.

At the species level (Figure 3C,F), the microbial composition exhibited a distinct pattern characterised by a concurrent decline in beneficial bacteria and a rise in potential pathobionts. Compared with the WT-C group, the WT-S.m, SHANK3-C, and SHANK3-S.m groups showed significantly reduced abundances of beneficial species such as *Bacteroides caecimuris*, *Bacteroides faecium*, *Bacteroides xylanisolvens*, and *Bacteroides thetaiotaomicron*, while the abundances of potential pathobionts including *Duncaniella dubosii*, *Muribaculum gordoncarteri*, and *Bacteroides uniformis* were significantly increased (*P* < 0.05). Furthermore, *S. mutans* gavage specifically enriched several bacterial species, such as *Parabacteroides merdae*, *Bacteroidales bacterium CF* (*P* < 0.05), *Phoenicibacter congonensis*, and *Mucinivorans hirudinis*, in both the WT-S.m and SHANK3-S.m groups, with a particularly enhanced abundance of *D. dubosii* observed in the SHANK3-S.m group.

### LEfSe analysis reveals group-specific biomarkers

**Figure 4.**
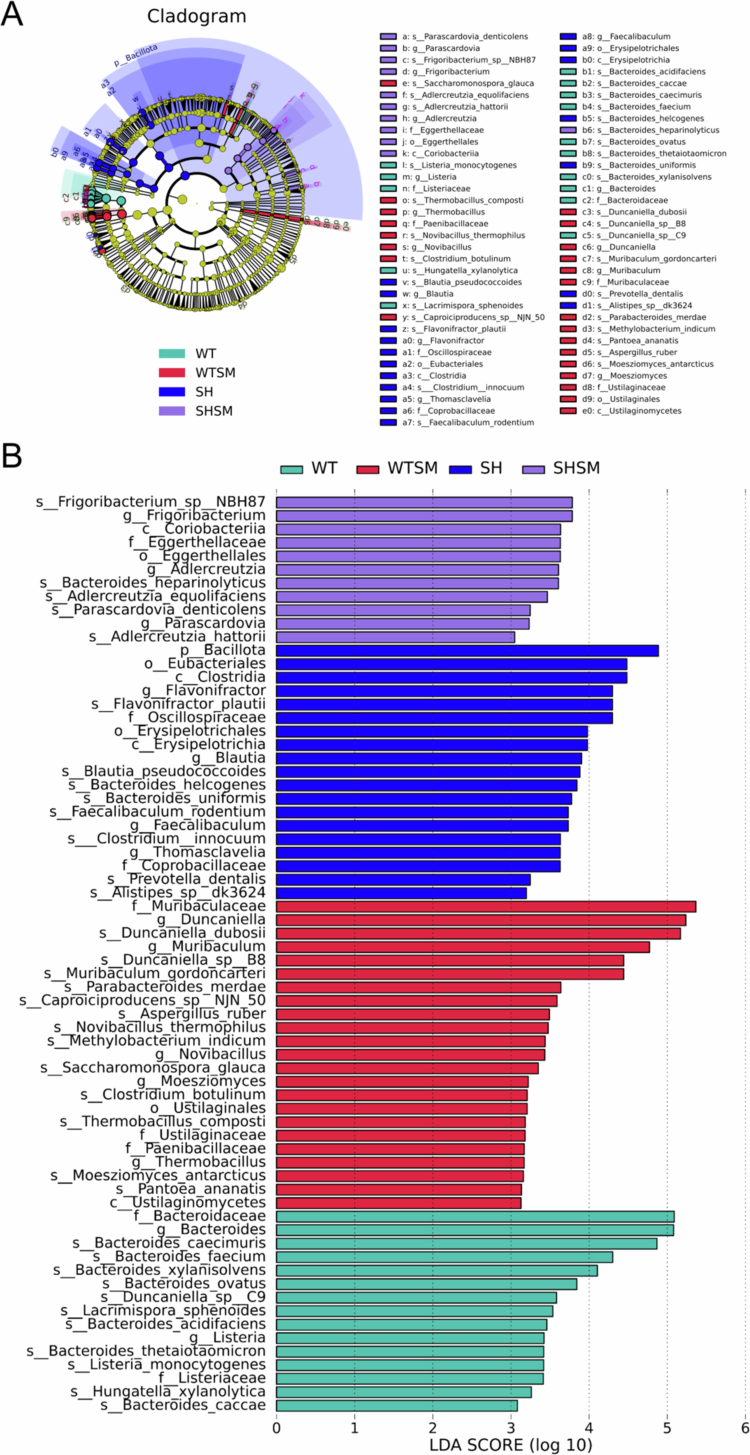
Identification of group-specific microbial biomarkers by LEfSe analysis. (A) Cladogram showing the phylogenetic distribution of bacterial taxa enriched in different groups. Colours represent taxa enriched in SHANK3-S.m (purple), SHANK3-C (blue), WT-S.m (red), and WT-C (green) groups, with yellow indicating non-significant taxa. The concentric circles represent taxonomic levels from domain to species. (B) Histogram of LDA scores showing the effect size of significantly different taxa among the four groups (LDA > 3, *P* < 0.05). Colours correspond to the respective enriched groups as in (A).

**Figure 5.**
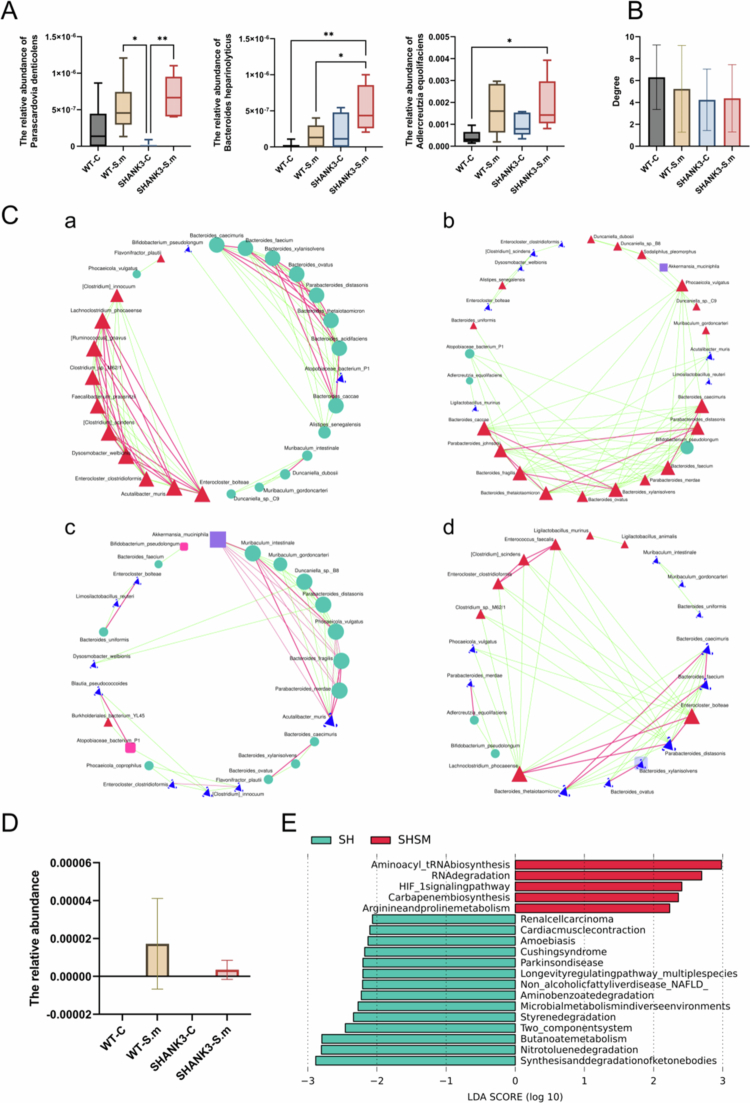
Microbial ecological networks and functional consequences of *S. mutans*colonisation. (A) Relative abundance of SHANK3-S.m group-specific bacterial biomarkers identified by LEfSe analysis. Data are presented as box plots; ^*^ *P* < 0.05, ^**^ *P* < 0.01. (B) Average network degree of gut microbial communities, indicating species interaction complexity across the four groups. (C) Co-occurrence networks of the top 30 abundant bacterial species. Red and green edges represent significant positive (r > 0) and negative (r < 0) correlations, respectively. Networks are shown for WT-C (a), WT-S.m (b), SHANK3-C (c), and SHANK3-S.m (d) groups. (D) Relative abundance of *S. mutans* in faecal samples from the four experimental groups. ^***^ *P* < 0.001. (E) Differential KEGG pathway activity (Level 3) between SHANK3-C and SHANK3-S.m groups.

To identify group-specific microbial biomarkers, we performed LEfSe analysis (LDA > 3, *P* < 0.05). The results are shown in Figures 4 and 5A. The SHANK3-S.m group was characterised by *Bacteroides heparinolyticus*, *Parascardovia denticolens*, and *Adlercreutzia hattorii*. Notably, the abundance of *P. denticolens* was significantly higher in the SHANK3-S.m group than in the SHANK3-C group (*P* < 0.05). *B. heparinolyticus* was significantly enriched in the SHANK3-S.m group compared to both WT groups, and *A. hattorii* was significantly increased in both *S. mutans*-gavaged groups (WT-S.m and SHANK3-S.m) relative to their non-gavaged controls (*P* < 0.05).

In contrast, the SHANK3-C group was enriched in species such as *Bacteroides uniformis* and *Prevotella dentalis*. The WT-S.m group was defined by biomarkers including *Duncaniella dubosii* and *Parabacteroides merdae*, while the WT-C group was predominantly associated with beneficial Bacteroides species like *B. caecimuris* and *B. thetaiotaomicron*.

### Co-occurrence network analysis indicates impaired microbiota stability in ASD model mice

By constructing species correlation networks, we found that the mean degree of the microbial networks was significantly higher in the WT-C and WT-S.m groups (6.3 and 5.2, respectively) than in the SHANK3-C and SHANK3-S.m groups (4.2 and 4.4, respectively) (Figure 5B), indicating weakened species interactions and reduced network stability in the ASD model.

Analysis of centrality metrics identified group-specific keystone taxa (Figure 5C). The WT-C network was characterised by beneficial Bacteroides species (e.g. *B. xylanisolvens*, *B. ovatus*), whereas the SHANK3-S.m network was dominated by *Enterocloster bolteae*. This pathobiont exhibited strong positive correlations with other pro-inflammatory species (e.g. *Parabacteroides distasonis* and *Lachnoclostridium phocaeense*, r = 1, *P* < 0.05) and negative correlations with beneficial species (e.g. *B. caecimuris*, *B. faecium*, and *B. thetaiotaomicron*, r = −0.94, *P* < 0.05), positioning it as a potential destabilising factor in the ASD gut ecosystem.

### S. mutans translocation triggers exacerbated dysbiosis in susceptible hosts

Metagenomic analysis confirmed successful translocation of orally administered *S. mutans* to the gut environment in both gavaged groups. Although the relative abundance of *S. mutans* showed a lower trend in SHANK3^-/-^ mice compared to WT controls (Figure 5D), the difference did not reach statistical significance, suggesting transient colonisation rather than stable persistence in the ASD gut.

### Significant alterations in gut microbiota functional profiles

**Figure 6.**
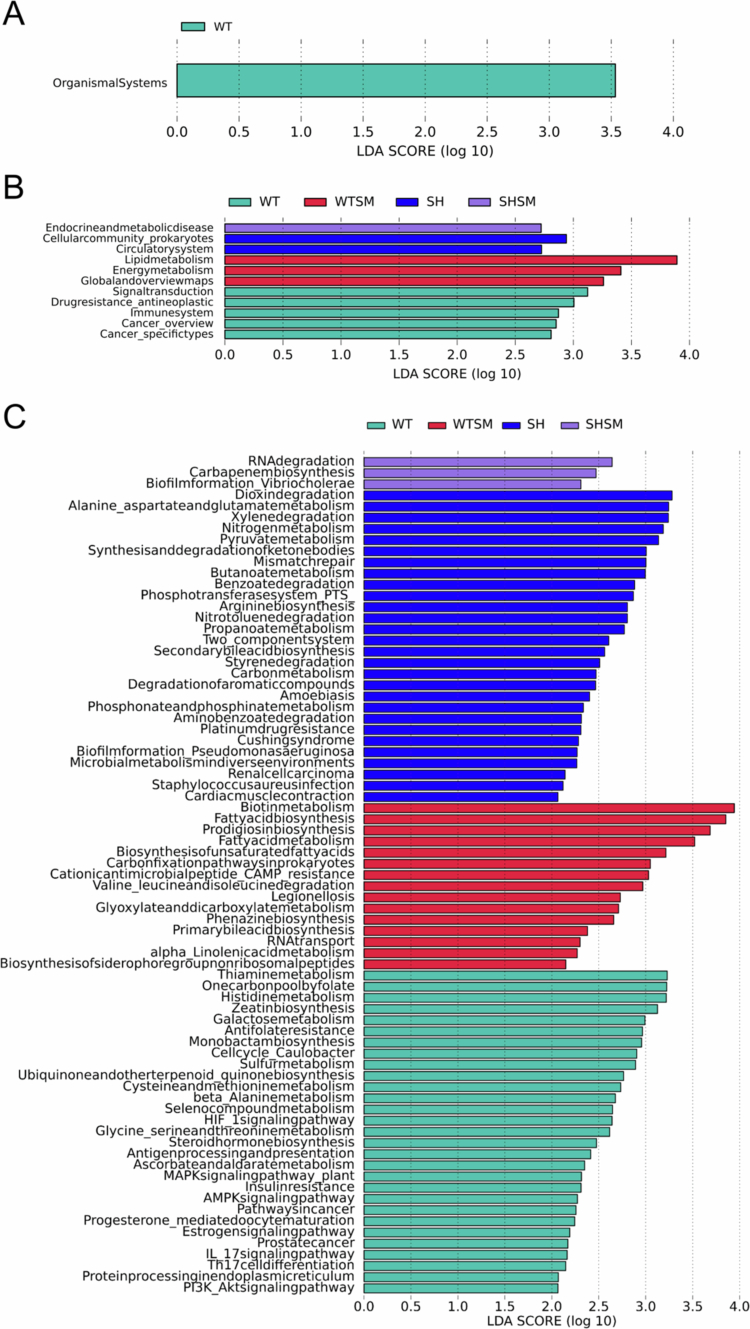
Differential functional enrichment of gut microbiota across KEGG pathway hierarchies. (A) Significantly enriched KEGG pathways at Level 1 between the four experimental groups (LDA > 2, *P* < 0.05). (B) Differential KEGG pathway abundance at Level 2, showing group-specific metabolic and functional preferences. (C) Distinct functional profiles at Level 3, revealing fine-scale metabolic alterations among the four groups.

KEGG functional enrichment analysis revealed extensive differences in the functional profiles of the gut microbiota among the four groups. At Level 2, the WT-C group was enriched for pathways related to the Immune system and Signal transduction, whereas the SHANK3-S.m group was specifically enriched for pathways associated with Endocrine and metabolic diseases (Figure 6B).

At the more detailed Level 3, we observed that the SHANK3-C group was significantly enriched in metabolic pathways crucial for neurological health, such as Butanoate metabolism and Synthesis and degradation of ketone bodies. However, in the SHANK3-S.m group, the activity of these pathways was reduced, with a shift towards enrichment in Aminoacyl-tRNA biosynthesis and the HIF-1 signalling pathway (Figures 5E, 6C).

## Discussion

This study establishes that oral administration of *S. mutans* enables gut translocation via the enteral route in SHANK3^-/-^ autism model mice. Despite comparable colonisation levels between genotypes, the ecological impact was severe in SHANK3^-/-^ mice, indicating that the ASD genetic background confers heightened susceptibility to microbiome disruption. These findings reveal a novel oral-gut axis mechanism in ASD pathophysiology, wherein transient pathobiont exposure triggers sustained dysbiosis in genetically susceptible hosts.

The SHANK3 gene, located on chromosome 22q13.3, encodes a master scaffolding protein enriched at the postsynaptic density of excitatory glutamatergic synapses. SHANK3 plays essential roles in synaptic organisation, dendritic spine maturation, and the regulation of glutamatergic signalling, thereby maintaining excitatory-inhibitory balance and supporting normal cognitive and social behaviours [36,37]. Mutations in SHANK3, including deletions, nonsense, and missense variants, are found in approximately 0.69% of individuals with ASD and are strongly associated with syndromic forms of ASD [38]. Consistent with its clinical relevance, SHANK3-deficient mouse models exhibit core ASD-like phenotypes, including social interaction deficits, repetitive behaviours, and altered synaptic structure and function [39,40]. These models provide a genetically and biologically relevant platform for investigating ASD pathophysiology, particularly in relation to synaptic dysfunction and systemic dysregulation.

Our results confirm that SHANK3 gene deletion alone induces significant alterations in gut microbial community structure (beta diversity), consistent with previous reports of gut ecological imbalance in both ASD individuals and model animals [11–13]. More importantly, we found that while *S. mutans* gavage induced unique microbial shifts in both WT and SHANK3^-/-^ mice, the ecological perturbation was more profound in the ASD model. This was characterised by a significant reduction in several species with probiotic potential, such as *Akkermansia muciniphila*, *Bacteroides caecimuris*, and *Bacteroides faecium*. These bacteria play crucial roles in maintaining intestinal barrier integrity [41,42], producing short-chain fatty acids (SCFAs) [43–46], and regulating neurotransmitters [47–50]. Conversely, several potential pathobionts associated with neurological and intestinal inflammatory disorders-including *Muribaculum gordoncarteri* [51], *Bacteroides uniformis* [52,53], and *Duncaniella dubosii*, were significantly enriched in *S. mutans*-treated and SHANK3-C groups. This pattern of ‘decreased beneficial bacteria and increased harmful bacteria’ collectively establishes an intestinal microenvironment in ASD model mice that is susceptible to invasion by oral pathogens.

**Figure 7.**
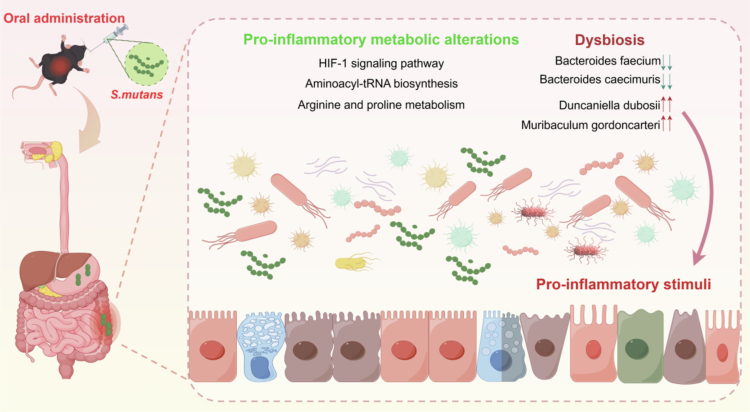
Schematic diagram illustrating the aggravation of gut dysbiosis and pro-inflammatory metabolic alterations in ASD model mice following oral gavage of *S. mutans*. This schematic summarises the key alterations in the gut microenvironment of ASD model mice after oral administration of *S. mutans*. The gavage of *S. mutans* exacerbates intestinal dysbiosis and ecological imbalance, characterised by an abnormal proliferation of specific bacterial species (including *Bacteroides faccium*, *Bacteroides caccimuris*, *Duncaniella dubosii*, and *Muribaculum gordoncarteri*). Concurrently, it triggers pro-inflammatory metabolic reprogramming, activating several key pathways such as the HIF-1 signalling pathway, aminoacyl-tRNA biosynthesis, and arginine and proline metabolism. These collective changes contribute to an enhanced pro-inflammatory state in the gut, which may subsequently influence host behaviour and neurodevelopment.

Species correlation network analysis further revealed the disruptive impact of *S. mutans* gavage on the gut microecology of ASD model mice. The lower average degree of the gut microbial network in SHANK3^-/-^ mice compared to WT mice indicated a pre-existing fragility in microbial community stability. Following *S. mutans* gavage, *Enterocloster bolteae*, as a pathogen frequently detected in children with ASD and implicated in neurotoxin metabolism [54,55], emerged as a central node in the SHANK3-S.m network. This bacterium showed significant negative correlations with several beneficial bacteria and positive correlations with pro-inflammatory taxa such as *Parabacteroides distasonis* and *Lachnoclostridium phocaeense* [56,57]. This suggests that *S. mutans* invasion may promote pro-inflammatory shifts and disrupt ecological balance by bolstering driver species like *E. bolteae*, thereby altering inherent interspecies interactions (Figure 7).

A key observation was that the relative abundance of *S. mutans* in faeces, while detectable in both gavaged groups, showed no significant enhancement in SHANK3^-/-^ mice compared to WT controls. This suggests that the gut environment of ASD model mice, potentially involving impaired gut barrier function, altered immune status, or specific ecological niches, is particularly vulnerable to *S. mutans*-induced ecological impacts rather than supporting enhanced colonisation. While a previous study reported failure to detect other oral streptococci in faeces of healthy mice after oral administration [31], our detection of *S. mutans* in both WT and ASD model mice confirms successful translocation. The more severe dysbiosis observed in SHANK3^-/-^ mice despite similar colonisation levels highlights the decisive role of host genetic background in determining ecological consequences rather than colonisation capacity. We speculate that the impaired intestinal barrier function commonly observed in ASD facilitates the pathological impact of translocated oral bacteria. This notion is supported by clinical observations in other gastrointestinal disorders. For instance, patients with IBD exhibit increased ectopic gut colonisation by oral-derived bacteria, such as Streptococcus and Prevotella species [58]. The prolonged persistence of orally administered bacteria in inflamed guts, as observed in our supporting data using a colitis model, aligns with the finding that the susceptible ASD gut microenvironment is more profoundly disrupted by *S. mutans*. Furthermore, Parascardovia denticolens, specifically enriched in the SHANK3-S.m group and known to enhance acid production and tolerance in *S. mutans* [59], might contribute to the exacerbated dysbiosis through interspecies interactions, rather than directly enhancing *S. mutans* colonisation.

In the SHANK3^-/-^ background, *S. mutans* intervention led to significant reprogramming of the gut microbial functional profile. Notably, pathways including aminoacyl-tRNA biosynthesis, arginine and proline metabolism, and the HIF-1 signalling pathway were significantly enriched in the SHANK3-S.m group compared to the SHANK3-C group. These pathways are implicated in various neurological disorders, including intracranial atherosclerosis [60], cerebral small vessel disease [61], depression [62,63], and Alzheimer's disease [64]. Specifically, the HIF-1 signalling pathway is activated in ASD-associated neuroinflammation [65], and arginine metabolism dysregulation has been reported in individuals with ASD [66,67]. These functional alterations suggest that *S. mutans* colonisation may not only disrupt microbial structure but also contribute to ASD-related neuroinflammation and metabolic abnormalities by affecting host-microbe co-metabolism, thereby acting as a second hit within the microbiota-gut-brain axis.

While our study demonstrates that *S. mutans* exacerbates gut dysbiosis in an ASD mouse model via the oral-gut axis, the translational relevance to human ASD warrants further investigation. This study has several limitations. First, although we observed correlations between *S. mutans* colonisation and changes in microbiota/behaviour, the direct causal links require further validation using approaches such as germ-free animals or antibiotic-mediated microbiota depletion. Second, the precise mechanisms by which *S. mutans* influences the host-whether through its own metabolites (e.g. imidazole propionate [68,69]) or indirectly by remodelling the entire gut microbial ecosystem-remain to be fully elucidated. Future studies will focus on the role of *S. mutans*-specific metabolites and investigate the specific pathways involved in gut barrier disruption and systemic immune activation. Furthermore, translating these preclinical findings to human populations necessitates targeted clinical investigations. Future research should quantify oral bacterial translocation in individuals with ASD and evaluate whether improved oral health interventions, such as caries prevention strategies or targeted antibacterial therapies, can alleviate gastrointestinal and behavioural symptoms. Additionally, longitudinal cohort studies integrating multi-omics profiling of both oral and gut microbiomes are needed to establish causal links between oral pathobionts and ASD progression.

## Conclusion

In summary, this study demonstrates that the oral cariogenic pathogen *S. mutans* can translocate to the gut and, despite showing no increased colonisation in SHANK3-deficient hosts, it provokes exacerbated gut microbiota dysbiosis in a genetically susceptible ASD mouse model. Key findings include the enrichment of pro-inflammatory and neuro-disorder-associated bacteria (e.g. *Duncaniella dubosii*, *Enterocloster bolteae*), a decline in beneficial SCFA-producing taxa, and a functional shift towards neuroinflammation-related metabolic pathways. These results underscore the critical role of the oral-gut axis in modulating gut ecology and systemic inflammation in ASD, offering a novel mechanistic perspective that links oral health to ASD-related gastrointestinal and behavioural pathophysiology. This work contributes to the growing body of evidence on the microbiota-gut-brain axis by highlighting oral pathogens as potential modulators of systemic and neural health in neurodevelopmental disorders, thereby proposing oral microbiome-targeted interventions as a complementary strategy for ASD management.

## Supplementary Material

Supplementary File Original Uncropped Gel Images.pptx Supplementary File Original Uncropped Gel Images.pptx

Supplementary file .docx Supplementary file .docx

## Data Availability

The datasets generated and/or analysed during the current study are available in the NCBI repository (PRJNA1364774).
