# Supplementary material for: Streptococcus mutans exacerbates gut microbiota dysbiosis in SHANK3 -/- autism model mice via the oral-gut axis
Source: J Oral Microbiol. 2026 Jun 4;18(1):2681259. doi: 10.1080/20002297.2026.2681259 (PMC13237793; doi:10.1080/20002297.2026.2681259)
Supplement: Supplementary File Original Uncropped Gel Images.pptx [file ZJOM_A_2681259_SM2063.pptx]

## Slide 1
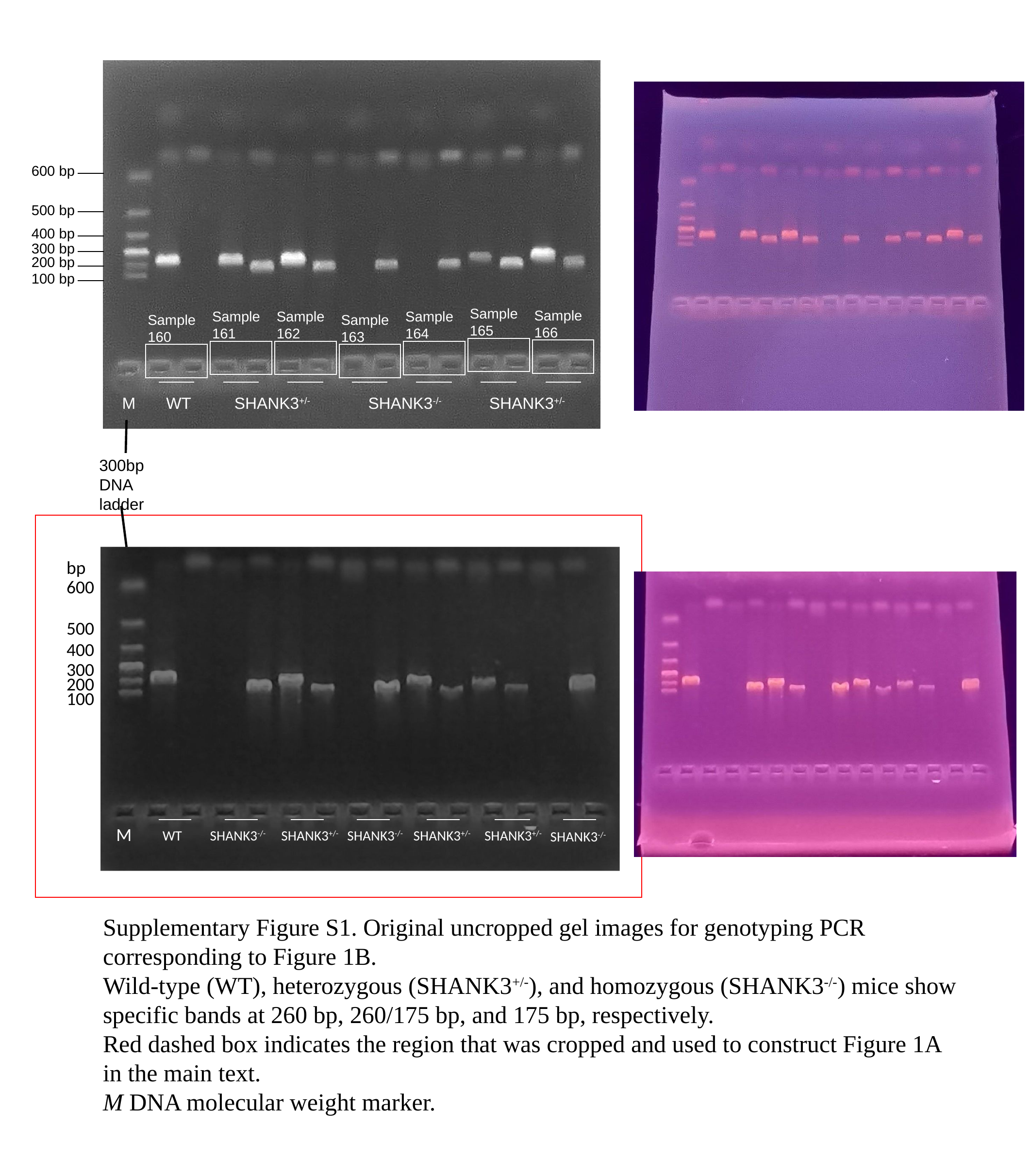

600 bp
500 bp
400 bp
300 bp
200 bp
100 bp
Sample 165
Sample 166
Sample 161
Sample 162
Sample 164
Sample 160
Sample 163
M
WT
SHANK3+/-
SHANK3-/-
SHANK3+/-
300bpDNA ladder
bp
600
500
400
300
200
100
M
WT
SHANK3-/-
SHANK3+/-
SHANK3-/-
SHANK3+/-
SHANK3+/-
SHANK3-/-
Supplementary Figure S1. Original uncropped gel images for genotyping PCR corresponding to Figure 1B.
Wild-type (WT), heterozygous (SHANK3+/-), and homozygous (SHANK3-/-) mice show specific bands at 260 bp, 260/175 bp, and 175 bp, respectively.
Red dashed box indicates the region that was cropped and used to construct Figure 1A in the main text.
M DNA molecular weight marker.
