# Supplementary material for: Streptococcus mutans exacerbates gut microbiota dysbiosis in SHANK3 -/- autism model mice via the oral-gut axis
Source: J Oral Microbiol. 2026 Jun 4;18(1):2681259. doi: 10.1080/20002297.2026.2681259 (PMC13237793; doi:10.1080/20002297.2026.2681259)
Supplement: Supplementary file .docx [file ZJOM_A_2681259_SM2064.docx]

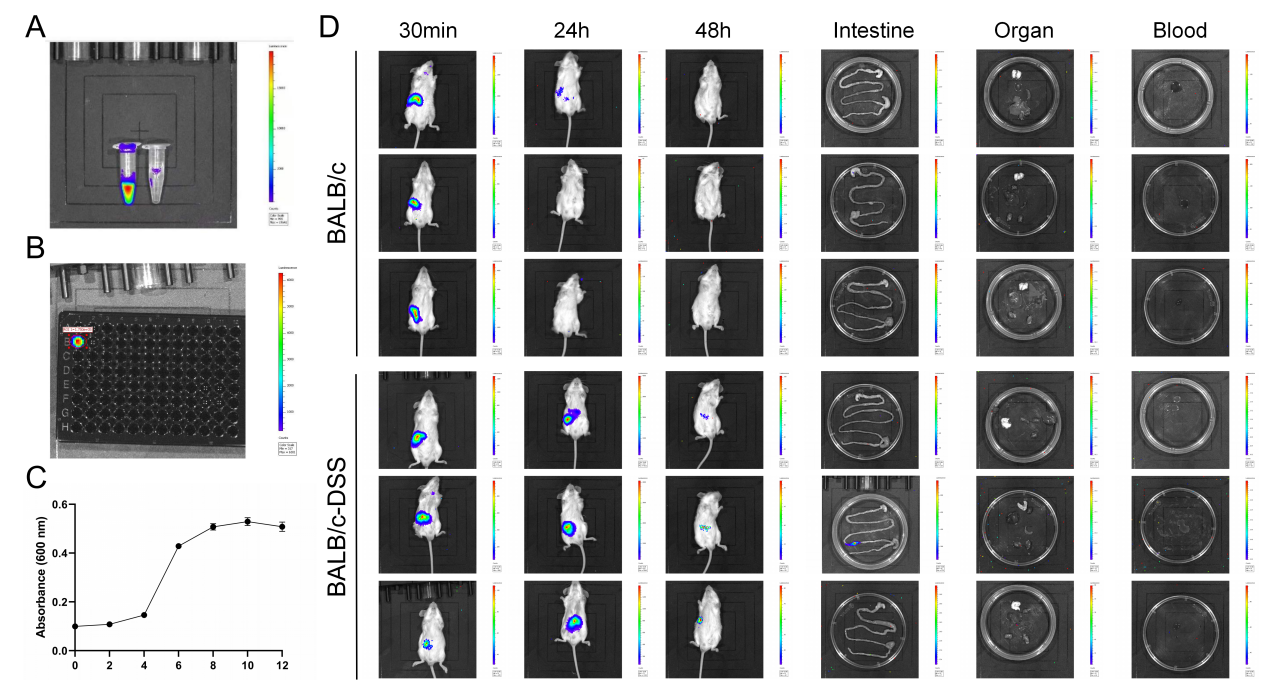


**Supplementary Figure 1. Validation of luciferase-tagged *S. mutans* (Luc-S.m) and its persistence in a DSS-induced colitis model.**

(A-C) *In vitro* characterization of Luc-S.m: fluorescence imaging in liquid culture (A) and microplate (B), and growth curve showing typical logarithmic and stationary phases (C). Luc-S.m maintained stable luciferase expression after serial passages.

(D) *In vivo* dynamics of Luc-S.m: Acute colitis was induced by administering 3% dextran sulfate sodium (DSS) in drinking water for 3 days. BALB/c mice with or without DSS-induced colitis were orally gavaged with Luc-S.m, and bioluminescence signals were monitored over 48 hours. Representative images show prolonged bacterial persistence in the guts of colitis mice compared to healthy controls at 24 and 48 hours post-gavage. The color scale indicates signal intensity from low (blue) to high (red).

**Supplementary Table 1. Sequencing results of the 16S rRNA of *S. mutans* UA159**

| SEQ UA159.27F.5066359.K4562A10.E12 909 DNA Composition 243 A; 185 C; 288 G; 193 T; 0 OTHER Percentage 26.7% A; 20.4% C; 31.7% G; 21.2% T; 0.0% OTHER MW(kDa) 282.95 ssDNA 560.42 dsDNA COLOURS sequence = 1 features = 0 | | | |
| --- | --- | --- | --- |
| 1 | GGGCTGGCGGCGTGCTATACATGCAAGTGGGACGCAAGGAGACACACTGT GCTTGCACAC | 481 | GGGACGGCTAACTACGTGCCAGCAGCCGCGGTAATACGTAGGTCCCGAGCGTTGTCCGGA |
| 61 | CGTGTTTTCTTGAGTCGCGAACGGGTGAGTAACGCGTAGGTAACCTGCCT ATTAGCGGGG | 541 | TTTATTGGGCGTAAAGGGAGCGCAGGCGGTCAGGAAAGTCTGGAGTAAAAGGCTATGGCT |
| 121 | GATAACTATTGGAAACGATAGCTAATACCGCATAATATTAATTATTGCATGATAATTGAT | 601 | CAACCATAGTGTGCTCTGGAAACTGTCTGACTTGAGTGCAGAAGGGGAGAGTGGAATTCC |
| 181 | TGAAAGATGCAAGCGCATCACTAGTAGATGGACCTGCGTTGTATTAGCTAGTTGGTAAGG | 661 | ATGTGTAGCGGTGAAATGCGTAGATATATGGAGGAACACCAGTGGCGAAAGCGGCTCTCT |
| 241 | TAAGAGCTTACCAAGGCGACGATACATAGCCGACCTGAGAGGGTGATCGGCCACACTGGG | 721 | GGTCTGTCACTGACGCTGAGGCTCGAAAGCGTGGGTAGCGAACAGGATTA GATACCCTGG |
| 301 | ACTGAGACACGGCCCAGACTCCTACGGGAGGCAGCAGTAGGGAATCTTCGGCAATGGACG | 781 | TAGTCCACGCCGTAAACGATGAGTGCTATGTGTTAGCCCTTTCCGGGGCTTAGTGCCGGA |
| 361 | AAAGTCTGACCGAGCAACGCCGCGTGAGTGAAGAAGGTTTTCGGATCGTA AAGCTCTGTT | 841 | GCTAACGCAATAAGCACTCCGCCTGGGGAGTACGACCGCAAGGTTGAAACTCAAAGGAAT |
| 421 | GTAAGTCAAGAACGTGTGTGAGAGTGGAAAGTTCACACAGTGACGGTAGCTTACCAGAAA | 901 | TGACGGGGG |

The 16S rRNA gene sequence of the isolate was analyzed using the NCBI BLAST tool, confirming its identity as *Streptococcus mutans* (GenBank: NR_042772.1) with 99% sequence similarity.

**Supplementary Table 2. Basic statistics of sample sequencing data**

| Group | Sample name | Raw reads | Clean reads | Cleaned (%) | Clean Q20 (%) | Clean Q30 (%) |
| --- | --- | --- | --- | --- | --- | --- |
| WT-C | WT1 | 26034421 | 24646148 | 94.67 | 99.17 | 97.14 |
|  | WT2 | 31103059 | 29032182 | 93.34 | 99.16 | 97.08 |
|  | WT3 | 25545178 | 24164124 | 94.59 | 99.2 | 97.19 |
|  | WT4 | 23181524 | 21854214 | 94.27 | 99.2 | 97.22 |
|  | WT5 | 29116767 | 27695889 | 95.12 | 99.2 | 97.23 |
|  | WT6 | 26465748 | 25186490 | 95.17 | 99.16 | 97.11 |
| WT-S.m | WTSM1 | 29744702 | 28612598 | 96.19 | 99.33 | 97.48 |
|  | WTSM2 | 22828658 | 22244754 | 97.44 | 99.39 | 97.55 |
|  | WTSM3 | 23867679 | 21373159 | 89.55 | 99.18 | 97.18 |
|  | WTSM4 | 37120905 | 30864772 | 83.15 | 99.18 | 97.13 |
|  | WTSM5 | 29887530 | 27051769 | 90.51 | 99.13 | 97 |
|  | WTSM6 | 30723639 | 28847854 | 93.89 | 99.13 | 96.98 |
| SHANK3-C | SH1 | 26881199 | 19119513 | 71.13 | 99.26 | 97.24 |
|  | SH2 | 33225984 | 29727390 | 89.47 | 99.36 | 97.57 |
|  | SH3 | 28870361 | 27156259 | 94.06 | 99.14 | 97.05 |
|  | SH4 | 28488369 | 26879612 | 94.35 | 99.14 | 97.04 |
|  | SH5 | 31452713 | 29722531 | 94.5 | 99.15 | 97.08 |
|  | SH6 | 27387121 | 26007395 | 94.96 | 99.21 | 97.2 |
| SHANK3-S.m | SHSM1 | 24896329 | 24146194 | 96.99 | 99.39 | 97.54 |
|  | SHSM2 | 21989602 | 21152205 | 96.19 | 99.32 | 97.43 |
|  | SHSM3 | 28727677 | 25280519 | 88 | 99.15 | 97.05 |
|  | SHSM4 | 26397503 | 24363313 | 92.29 | 99.12 | 96.94 |
|  | SHSM5 | 27883429 | 26103700 | 93.62 | 99.08 | 96.83 |
|  | SHSM6 | 31067309 | 27776684 | 89.41 | 99.13 | 97.02 |

Raw reads: number of sequencing raw reads; Clean reads: number of high-quality reads; Cleaned (%): percentage of reads remaining after filtering; Clean Q20 (%): proportion of bases with quality score > 20; Clean Q30 (%): proportion of bases with quality score > 30.
